# Supplementary figures and images for: The pharmacodynamic and differential gene expression analysis of PPAR α/δ agonist GFT505 in CDAHFD-induced NASH model
Source: PLoS One. 2020 Dec 16;15(12):e0243911. doi: 10.1371/journal.pone.0243911 (PMC7743980; doi:10.1371/journal.pone.0243911)

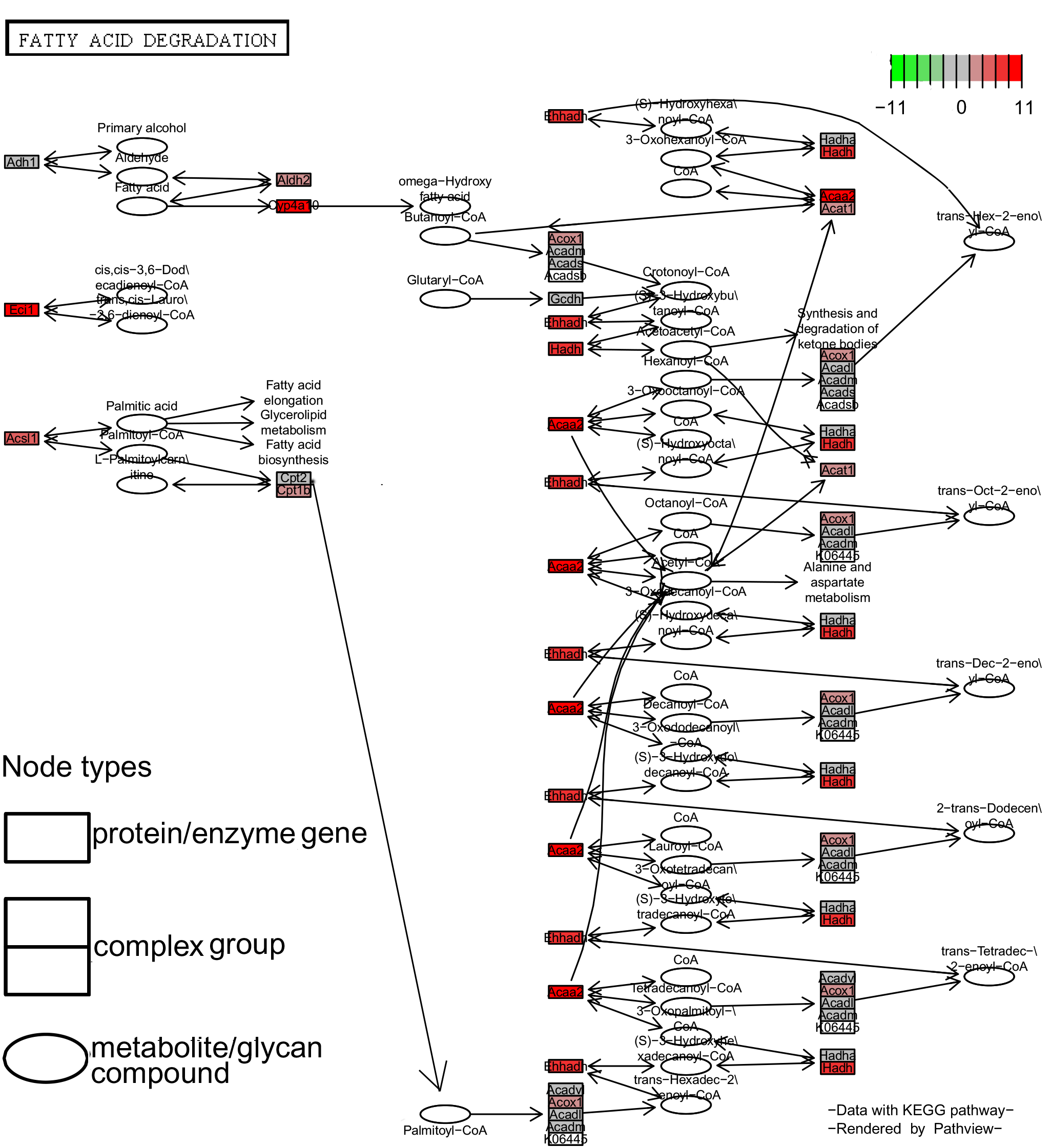

Supplement: S1 Fig — (TIF) [file pone.0243911.s001.tif]

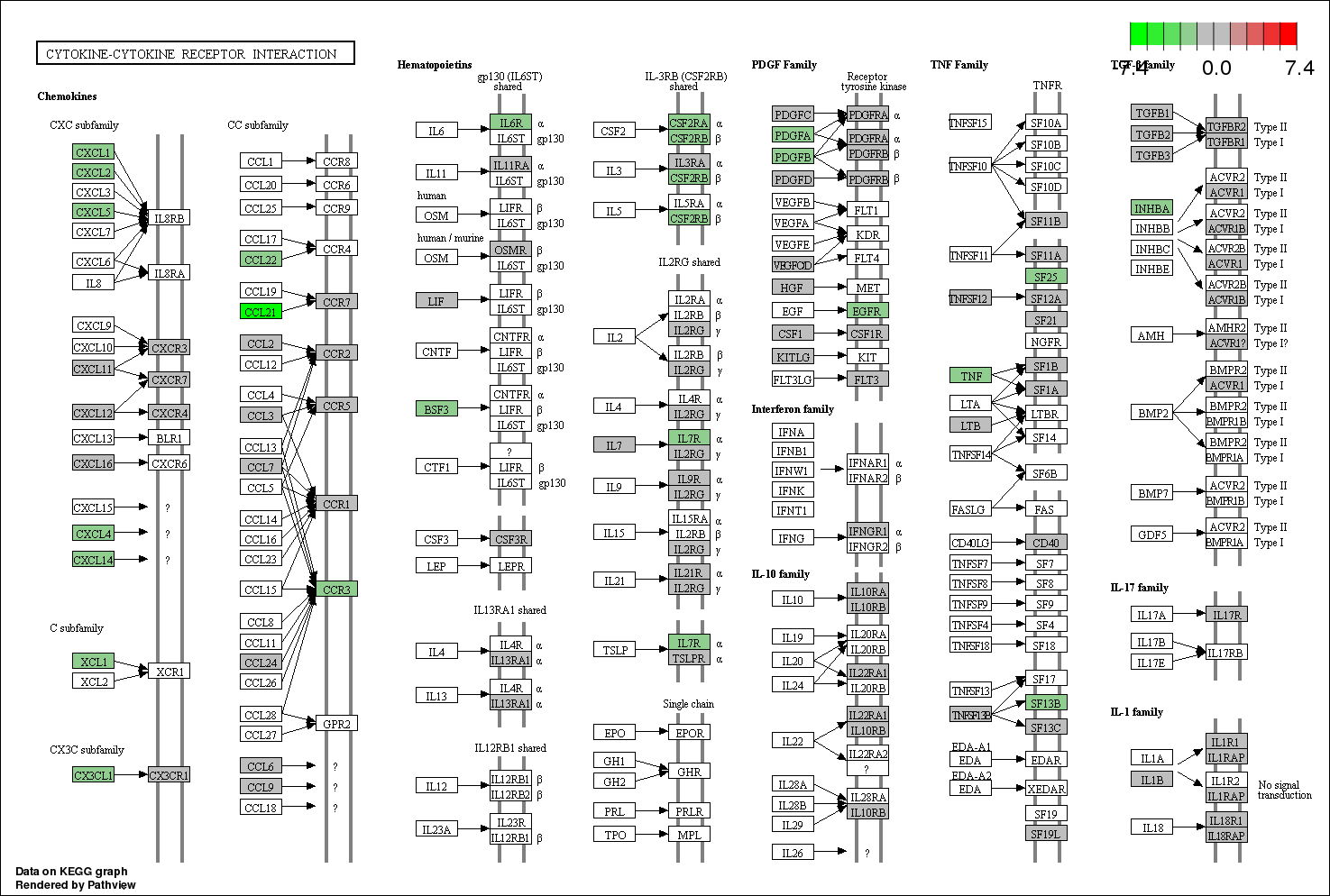

Supplement: S2 Fig — (TIF) [file pone.0243911.s002.tif]
