# Supplementary material for: The pharmacodynamic and differential gene expression analysis of PPAR α/δ agonist GFT505 in CDAHFD-induced NASH model
Source: PLoS One. 2020 Dec 16;15(12):e0243911. doi: 10.1371/journal.pone.0243911 (PMC7743980; doi:10.1371/journal.pone.0243911)
Supplement: S1 Table — (DOC) [file pone.0243911.s003.doc]

**S1 Table. GFT**505 treatment most significantly affect 10 Biological Process by Gene Ontology enrichment analysis.

| **Biological Process** | | **BgRatio^a^** | | **padj^b^** | | **geneID** | |
| --- | --- | --- | --- | --- | --- | --- | --- |
| cofactor metabolic process | 315/16729 | | 7.25E-24 | | Acot1,Acot2,Ehhadh,Pdk4,Me1,Acacb,Hsd17b4,Gpd2,Crot,Vnn1,Acot8,Ces1d,Mdh2,Hsd11b1,Pkm,Gpd1,Acaa2,Suclg2,Dlst,Acss1,Acot5,Hmgcl,Acot12,Hk3,Slc37a2,Acot6,Vcp,Ncf2,Pank1,Pcbd1,Acot4,Acot7,Mdh1,Ndufa9,Coq4,Fpgs,Pgam1,Acaca,Acot3,Fbp1,Cox10 | |  |
| fatty acid metabolic process | 344/16729 | | 7.30E-20 | | Fabp3,Acot1,Acot2,Fabp4,Ehhadh,Pdk4,Fabp1,Scd1,Acaa1b,Slc27a1,Cyp2a22,Ucp3,Dbi,Cyp2b13,Mgll,Hadhb,Acacb,Ech1,Decr1,Acsl5,Ephx2,Crat,Acadm,Eci1,Hadha,Acadl,Acad10,Acad12,Acox1,Cpt2,Acad11,Abcd3,Cyp2j5,Fabp2,Anxa1,Acsf3,Apoa2,Acaa2,Acot12,Cyp4a10,Hadh,Acaa1a,Agmo,Fam213b,Adipor2,Gpat4,Acads,Mif,Ncf1,Decr2,Acot4,Apoa4,Acaca,Acox2 | |  |
| coenzyme metabolic process | 266/16729 | | 7.30E-20 | | Acot1,Acot2,Ehhadh,Pdk4,Me1,Acacb,Hsd17b4,Gpd2,Crot,Vnn1,Acot8,Ces1d,Mdh2,Hsd11b1,Pkm,Gpd1,Acaa2,Suclg2,Dlst,Acss1,Acot5,Hmgcl,Acot12,Acot6,Acot4,Acot7,Acaca,Dcxr,Mpc2,Gpam,Prkaa2,Acot3 | |  |
| organic acid catabolic process | 163/16729 | | 3.33E-15 | | Acot2,Ehhadh,Fabp1,Dbi,Hadhb,Decr1,Acsl5,Acadm,Eci1,Hadha,Csad,Acadl,Acad10,Acad12,Hsd17b4,Ces1f,Crot,Acadvl,Lonp2,Acot8,Eci2,Acox1,Etfdh,Ces1d,Acad11,Abcd3,Hacl1,Acsf3,Slc27a4,Lipe,Acaa2,Acox2 | |  |
| carboxylic acid catabolic process | 163/16729 | | 3.33E-15 | | Acot2,Ehhadh,Fabp1,Dbi,Hadhb,Decr1,Acsl5,Acadm,Eci1,Hadha,Csad,Acadl,Acad10,Acad12,Hsd17b4,Ces1f,Crot,Acadvl,Lonp2,Acot8,Eci2,Acox1,Etfdh,Ces1d,Acad11,Abcd3,Hacl1,Acsf3,Slc27a4,Lipe,Acaa2,Acox2 | |  |
| small molecule biosynthetic cccprocess | 429/16729 | | 4.73E-15 | | Fabp3,Pdk4,Scd1,Serpina12,Fbp2,Slc27a1,Mgll,Acacb,Acadm,Acadl,Pltp,Elovl3,Gpd2,Acadvl,Lepr,Rdh16,G6pc,Chpt1,Abcd3,Ndufab1,Anxa1,Acsf3,Apoa2,Pkm,Chkb,Gpd1,Elovl5,Hmgcs2,Acss1,Hmgcl,Thnsl2,Rbp1,Sec14l2,Agmo,Fam213b,Gpat4,Mif,Park7,Atf3,Decr2 | |  |
| cellular respiration | 120/16729 | | 1.18E-14 | | Mdh2,Cox8a,Gpd1,Suclg2,Dlst,Idh1,Slc37a2,Ndufa10,Uqcrb,Vcp,Cat,Aco2,Ndufa5,Cisd1,Cycs,ND2,Park7,Sod2,Cox8b,Uqcrc1,Uqcr10,Sucla2,Mdh1,Uqcrc2,Pmpcb,Dlat,Ndufs1,Trap1,Dld,Suclg1,Ndufs6,Cox5a,Sdha,Coq9,Ndufs4,Sdhc | |  |
| generation of precursor metabolites and energy | 271/16729 | | 2.55E-14 | | Acadm,Crot,Acox1,Etfdh,G6pc,Mdh2,Pkm,Ndufb3,Cox8a,Gpd1,Suclg2,Dlst,Idh1,Slc25a33,Hk3,Chchd10,Slc37a2,Ndufa10,Uqcrb,Vcp,Cat,Aco2,Ndufa5,Cisd1,Cycs,ND2,Mif,Park7,Sod2,Cox8b | |  |
| lipid biosynthetic process | 481/16729 | | 3.15E-13 | | Fabp3,Hsd17b11,Pdk4,Scd1,Mogat1,Serpina12,Slc27a1,Dbi,Abhd6,Mgll,Acacb,Acsl5,Sptlc3,Pla2g6,Acadl,Elovl3,Acadvl,Fitm1,Kdsr,Gal3st1,Rdh16,Hsd17b12,Chpt1,Abcd3,Ndufab1,Anxa1,Acsf3,Apoa2,Hsd11b1,Agpat9,Chkb,Elovl5,Hmgcs2,Acss1,Acsl1,Thnsl2,Idh1,Lpcat3,Rbp1,Sec14l2,Agmo,Fam213b,Gpat4,Agpat2,Mif,Decr2,Wdtc1,Pnpla8,Acot7 | |  |
| small molecule catabolic process | 235/16729 | | 7.15E-13 | | Acot2,Ehhadh,Fabp1,Dbi,Hadhb,Decr1,Acsl5,Acadm,Eci1,Hadha,Csad,Acadl,Acad10,Acad12,Hsd17b4,Ces1f,Crot,Acadvl,Lonp2,Acot8,Eci2,Acox1,Etfdh,Adh1,Ces1d,Glo1,Acad11,Abcd3,Hacl1,Acsf3,Slc27a4,Lipe,Acaa2,Thnsl2,Hadh,Acot4,Acot7,Acox2 | |  |
| ^a^:BgRatio, M/N, M: the genes in the pathway, N: the genes in GenBank | | | | | | |  |
| ^b^:padj: adjusted p-value | | | | | | |  |
