# Supplementary material for: The pharmacodynamic and differential gene expression analysis of PPAR α/δ agonist GFT505 in CDAHFD-induced NASH model
Source: PLoS One. 2020 Dec 16;15(12):e0243911. doi: 10.1371/journal.pone.0243911 (PMC7743980; doi:10.1371/journal.pone.0243911)
Supplement: S2 Table — (DOC) [file pone.0243911.s004.doc]

**S2 Table. GFT505 treatment most significantly affect 10 Process by Reactom enrichment analysis.**

| **Description** | **BgRatio^a^** | **padj^b^** | **geneID** |
| --- | --- | --- | --- |
| The citric acid (TCA) cycle and respiratory electron transport | 142/5907 | 3.45E-27 | Pdk4,Ucp3,Etfdh,Ndufab1,Mdh2,Atp5g3,Ndufb3,Cox8a,Suclg2,ND1,Dlst,Atp5b,Ndufa10,Uqcrb,Cox6a1,Cox6b1,Atp5d,Aco2,Uqcrfs1,Ndufa5,Pdp2,Atp5a1,Cycs,ND2 |
| Respiratory electron transport, ATP synthesis by chemiosmotic coupling, and heat production by uncoupling proteins. | 106/5907 | 1.16E-22 | Ucp3,Etfdh,Ndufab1,Atp5g3,Ndufb3,Cox8a,ND1,Atp5b,Ndufa10,Uqcrb,Cox6a1,Cox6b1,Atp5d,Uqcrfs1,Ndufa5,Atp5a1,Cycs,ND2,Ndufv3,Cox5b,LOC102641600,Uqcrc1,Uqcr10,Atp5f1,Ndufa9,Cox5a,Sdha,Cox7b |
| Respiratory electron transport | 83/5907 | 8.71E-20 | Etfdh,Ndufab1,Ndufb3,Cox8a,ND1,Ndufa10,Uqcrb,Cox6a1,Cox6b1,Uqcrfs1,Ndufa5,Cycs,ND2,Ndufv3,Cox5b,LOC102641600,Uqcrc1,Uqcr10,Cox5a,Sdha,Ndufa12,Ndufs4,Sdhc,Ndufa4,Cyc1,Ndufv1,Ndufs7,Cox7c,Cox7b,COX3,COX1,Cox7a2l |
| Metabolism of lipids and lipoproteins | 491/5907 | 6.12E-15 | Cyp4a14,Fabp3,Fabp4,Fabp1,Cyp4a31,Cyp4a12a,Acaa1b,Cyp4a12b,Mgll,Hadhb,Acacb,Decr1,Acsl5,Ephx2,Crat,Acadm,Eci1,Sptlc3,Pla2g6,Hadha,Acadl,Cyp4a32,Hsd17b4,Elovl3,Gpd2,Crot,Acadvl,Kdsr,Acot8,Acox1,Cyp2c29,Hsd17b12,Mttp,Amn,Cpt2,Fabp2,Apoa2,Lpcat3,Hadh,Acaa1a,Fam213b,Pi4k2b,Fabp7,Gpat4,Acads,Abcc1,Pik3r5 |
| Mitochondrial translation elongation | 82/5907 | 6.97E-13 | Gfm1,Mrpl38,Tufm,Mrpl14,Mrps5,Mrpl50,Mrpl17,Mrpl13,Mrps10,Mrpl51,Mrps12,Mrpl16,Mrpl49,Mrpl43,Mrpl15,Mrpl10,Mrpl37,Mrps35,Mrpl9,Mrps27,Mrps18b,Mrpl2,Mrpl39,Mrpl42,Ptcd3,Mrps7 |
| Mitochondrial translation | 87/5907 | 1.68E-12 | Gfm1,Mrpl38,Tufm,Gfm2,Mrpl14,Mrps5,Mrpl50,Mrpl17,Mtif2,Mrpl13,Mrps10,Mrpl51,Mrps12,Mrpl16,Mrpl49,Mrpl43,Mrpl15,Mrpl10,Mrpl37,Mrps35,Mrpl9,Mrps27,Mrps18b,Mrpl2,Mrpl39,Mrpl42 |
| Mitochondrial translation termination | 82/5907 | 4.07E-12 | Mrpl38,Gfm2,Mrpl14,Mrps5,Mrpl50,Mrpl17,Mrpl13,Mrps10,Mrpl51,Mrps12,Mrpl16,Mrpl49,Mrpl43,Mrpl15,Mrpl10 |
| Mitochondrial translation initiation | 81/5907 | 6.59E-12 | Mrpl38,Mrpl14,Mrps5,Mrpl50,Mrpl17,Mtif2,Mrpl13,Mrps10,Mrpl51,Mrps12,Mrpl16,Mrpl49,Mrpl43,Mrpl15,Mrpl10,Mrpl37,Mrps35,Mrpl9,Mrps27,Mrps18b |
| Fatty acid, triacylglycerol, and ketone body metabolism | 87/5907 | 3.92E-08 | Fabp1,Hadhb,Acacb,Decr1,Acsl5,Acadm,Eci1,Hadha,Acadl,Elovl3,Gpd2,Acadvl,Hsd17b12,Cpt2,Agpat9,Gpd1,Acat1,Elovl5,Slc25a20,Hmgcs2,Hmgcl,Acsl1,Hadh,Gpat4,Acads,Agpat2,Pcca,Tecr,Lclat1,Acaca,Gpam,Prkaa2,Lpin2,Hacd1,Cpt1b,Oxct1 |
| Metabolism of amino acids and derivatives | 184/5907 | 6.30E-08 | Csad,Suox,Grhpr,Slc25a10,Ckb,Acat1,Dlst,Slc6a8,Pcbd1,Bckdha,Aadat,Mccc2,Aldh9a1,Hibch,Psmb7,Tdo2,Got2,Dld |
| ^a^:BgRatio, M/N, M: the genes in the pathway, N: the genes in GenBank | | | |
| ^b^:padj: adjusted p-value | | | |
