# Supplementary material for: The pharmacodynamic and differential gene expression analysis of PPAR α/δ agonist GFT505 in CDAHFD-induced NASH model
Source: PLoS One. 2020 Dec 16;15(12):e0243911. doi: 10.1371/journal.pone.0243911 (PMC7743980; doi:10.1371/journal.pone.0243911)
Supplement: S3 Table — (DOC) [file pone.0243911.s005.doc]

**S3 Table. GFT505 treatment most significantly affect 10 Pathways by Kyoto Encyclopedia of Genes and Genomes enrichment analysis.**

| **Description** | **BgRatio^a^** | **padj^b^** | **geneID** |
| --- | --- | --- | --- |
| Metabolic pathways | 1106/5208 | 1.64E-18 | Cyp4a14,Ehhadh,Cyp4a31,Cyp4a12a,Cox6b2,Acaa1b,Aldh3a2,Cyp4a12b,Fbp2,Me1,Cyp2b13,Mgll,Hadhb,Acacb,Acsl5,Ephx2,Acadm,Sptlc3,Pla2g6,Hadha,Csad,Acadl,Ugt2b1,Cyp4a32,Ces1e,Hsd17b4,Acadvl,Kdsr,Gal3st1,Rdh16,Acox1,Cyp2c29,Uck1,Hsd17b12,Adh1,G6pc,Grhpr,Ces1d,Dhrs4,Atp6v0d2,Chpt1,Cyp2j5,Ndufab1,Tymp,Mdh2,Acss3,Hsd11b1,Agpat9,Pnliprp1,Atp5g3,Cyp2c38,Pkm,Chkb,Ndufb3,Cox8a,Ckb,Pafah2,Acat1,Acat2,Acaa2,Suclg2,ND1,Dlst,Hmgcs2,Acss1 |
| Parkinson's disease | 130/5208 | 1.21E-14 | Cox6b2,Ndufab1,Atp5g3,Ndufb3,Cox8a,ND1,Atp5b,Ndufa10,Uqcrb,Cox6a1,Cox6b1,Atp5d,Uqcrfs1,Ndufa5,Atp5a1,Cycs,ND2,Ndufv3,Park7,Cox5b,Cox8b,Uqcrc1,Uqcr10,Atp5f1,Ndufa9,Uqcrc2,Ndufa3,Ndufs1 |
| Oxidative phosphorylation | 129/5208 | 8.39E-12 | Cox6b2,Atp6v0d2,Ndufab1,Atp5g3,Ndufb3,Cox8a,ND1,Atp5b,Ndufa10,Uqcrb,Cox6a1,Cox6b1,Atp5d,Uqcrfs1,Ndufa5,Tcirg1,Atp5a1,ND2,Ndufv3,Cox5b,Cox8b,Uqcrc1,Uqcr10,Ppa2,Atp5f1,Ndufa9,Uqcrc2,Ndufa3,Ndufs1,Cox7a1,Ndufb10,Ndufs2,Ndufs3,Uqcr11,Ndufb11,Ndufc1,Cox6c,Atp5c1,Atp5o,Ndufs6,Cox5a,Cox10 |
| Alzheimer's disease | 169/5208 | 2.61E-09 | Cox6b2,Ndufab1,Atp5g3,Ndufb3,Cox8a,Bace1,Atp5b,Ndufa10,Uqcrb,Cox6a1,Cox6b1,Atp5d,Uqcrfs1,Ndufa5,Atp5a1,Cycs,Ndufv3,Cox5b,Cox8b,Uqcrc1,Uqcr10,Atp5f1,Ndufa9,Uqcrc2,Ndufa3,Ndufs1,Cox7a1,Ndufb10 |
| Huntington's disease | 177/5208 | 4.54E-08 | Cox6b2,Ndufab1,Atp5g3,Ndufb3,Cox8a,Atp5b,Ndufa10,Uqcrb,Cox6a1,Cox6b1,Atp5d,Uqcrfs1,Ndufa5,Atp5a1,Cycs,Ndufv3,Sod2,Cox5b,Cox8b,Uqcrc1,Uqcr10,Atp5f1,Ndufa9,Uqcrc2,Ndufa3,Ndufs1,Cox7a1,Ndufb10,Ndufs2,Ndufs3,Sod1,Uqcr11,Gpx1,Ndufb11,Ndufc1,Cox6c,Hip1,Atp5c1,Atp5o,Ndufs6,Cox5a |
| Peroxisome | 79/5208 | 9.71E-08 | Ehhadh,Acaa1b,Ech1,Acsl5,Ephx2,Crat,Hsd17b4,Crot,Pex11a,Acot8,Eci2,Acox1,Dhrs4,Abcd3,Hacl1,Gstk1,Pxmp4,Hmgcl,Acsl1,Idh1,Acaa1a,Cat,Sod2,Decr2,Pex13,Slc27a2,Mlycd,Pex16,Sod1,Nudt12,Pex1,Pex19,Pex3,Pmvk,Acox2,Scp2,Pex7,Hao1,Pex5,Acsl3,Idh2,Pex14,Abcd2,Gnpat,Far1,Amacr,Prdx1,Paox,Phyh,Pex6,Pecr,Ddo,Prdx5,Pex11g,Slc25a17,Agxt,Mpv17,Mpv17l,Nudt19,Agps,Far2,Pex12,Pex11b,Baat,Pxmp2 |
| Proteasome | 44/5208 | 2.36E-06 | Psmb7,Psmd1,Psmd4,Psmc1,Psmd11,Psmb3,Psmd2,Psmd3,Psma1,Psmf1,Psma4,Psmc3,Psmb1,Psmc4,Psmb4,Psma5,Psma6,Psmb5,Psmd13,Pomp,Psmd12,Psma3,Psmb2,Psme1,Psmc6,Psmd7,Psmc2,Psmd14,Psmd6,Psma2,Psme4,Shfm1,Psma7,Psmb8,Psmb6,Psmc5,Psmb10,Psme2,Psmd8 |
| PPAR signaling pathway | 73/5208 | 2.13E-05 | Cyp4a14,Fabp3,Fabp4,Ehhadh,Fabp1,Cyp4a31,Scd1,Cyp4a12a,Acaa1b,Cyp4a12b,Slc27a1,Me1,Dbi,Acsl5,Acadm,Acadl,Cyp4a32,Pltp,Angptl4,Acox1,Cpt2,Fabp2,Aqp7,Apoa2,Slc27a4,Hmgcs2,Acsl1,Cyp4a10,Acaa1a,Fabp7,Slc27a2,Cd36,Acox2,Scp2,Cpt1b,Acsl3,Ubc,Sorbs1,Fads2,Gk,Cyp8b1,Ppara,Slc27a5,Rxrg,Cyp27a1,Pck2,Fabp5,Pdpk1,Cyp7a1,Cpt1c,Lpl,Rxrb,Olr1,Apoa5,Slc27a6,Plin1,Pck1 |
| Citrate cycle (TCA cycle) | 29/5208 | 2.13E-05 | Mdh2,Suclg2,Dlst,Idh1,Aco2,Sucla2,Mdh1,Dlat,Dld,Suclg1,Sdha,Sdhc,Cs,Pdha1,Ogdh,Idh3b,Pdhb,Idh3g,Idh2,Sdhd,Pcx,Fh1,Aco1,Pck2,Sdhb,Acly,Pck1 |
| Fatty acid degradation | 45/5208 | 3.68E-05 | Cyp4a14,Ehhadh,Cyp4a31,Cyp4a12a,Acaa1b,Aldh3a2,Cyp4a12b,Hadhb,Acsl5,Acadm,Eci1,Hadha,Acadl,Cyp4a32,Acadvl,Eci2,Acox1,Adh1,Cpt2,Acat1,Acat2,Acaa2,Acsl1,Cyp4a10,Hadh,Acaa1a,Acads,Aldh9a1,Cpt1b,Aldh1a1,Acsl3,Echs1,Gcdh,Aldh7a1,Adh5,Aldh2,Cpt1c,Aldh1b1 |
| ^a^:BgRatio, M/N, M: the genes in the pathway, N: the genes in GenBank | | | |
| ^b^:padj: adjusted p-value | | | |
