# Supplementary material for: The pharmacodynamic and differential gene expression analysis of PPAR α/δ agonist GFT505 in CDAHFD-induced NASH model
Source: PLoS One. 2020 Dec 16;15(12):e0243911. doi: 10.1371/journal.pone.0243911 (PMC7743980; doi:10.1371/journal.pone.0243911)
Supplement: S4 Table — (DOC) [file pone.0243911.s006.doc]

**S4 Table. The change of Lipid metabolism related genes, inflammation reaction related genes and fibrosis related genes expression.**

| **Gene** | **Fold change（RNA-SEQ）** | **Fold change（RT-PCR）** | **Gene Description** |
| --- | --- | --- | --- |
| Pparα | 1.87 | 0.98 | Peroxisome proliferator activated receptor alpha |
| Acox1 | 7.43 | 6.80 | Peroxisomal acyl-coenzyme A oxidase 1 |
| Cpt1b | 10.03 | 56.31 | Carnitine palmitoyltransferase 1b |
| Fabp4 | 22.83 | 30.44 | Fatty acid binding protein 4 |
| Ehhadh | 63.95 | 105.33 | Enoyl CoA hydratase/3-hydroxyacyl CoA |
| Fabp3 | 416.09 | 567.48 | Fatty acid binding protein 3 |
| Ccl6 | 0.37 | 0.08 | Chemokine (C-C-motif) ligand 6 |
| Ccl9 | 0.37 | 0.28 | Chemokine (C-C-motif) ligand 9 |
| Cxcl14 | 0.23 | 0.15 | chemokine_(C-X-C_motif)_ligand_14 |
| Pik3r5 | 0.26 | 0.26 | Phosphoinositide 3 kinase regulatory Subunit 5 |
| PDGFα | 0.31 | 0.10 | Platelet derived growth factor alpha |
| PDGFβ | 0.35 | 0.11 | Platelet derived growth factor B |
| TFGβ2 | 0.41 | 0.09 | Transforming growth factor beta 2 |
| TGFβ1 | 0.51 | 0.14 | Transforming growth factor beta 1 |
| Timp2 | 0.40 | 0.09 | Tissue inhibitor of metalloproteinase 2 |
| Timp1 | 0.28 | 0.36 | Tissue inhibitor of metalloproteinase 1 |
| Lamc3 | 0.29 | 0.07 | laminin gamma 3 |
| Col3a1 | 0.42 | 0.11 | Collagen type III alpha 1 |
| Col1a2 | 0.43 | 0.10 | Collagen type I alpha 2 |
| Col1a1 | 0.44 | 0.05 | Collagen type I alpha 1 |
| Hapln4 | 0.45 | 0.16 | Hyaluronan acid and proteoglycan link protein 4 |
| Timp3 | 0.70 | 0.24 | Tissue inhibitor of metalloproteinase 3 |
