# Supplementary material for: The pharmacodynamic and differential gene expression analysis of PPAR α/δ agonist GFT505 in CDAHFD-induced NASH model
Source: PLoS One. 2020 Dec 16;15(12):e0243911. doi: 10.1371/journal.pone.0243911 (PMC7743980; doi:10.1371/journal.pone.0243911)
Supplement: S5 Table — (DOC) [file pone.0243911.s007.doc]

**S5 Table. Primers sequences used for RT-PCR.**

| **Gene name** | **Primer name** | **sequence(5＇to 3＇)** |
| --- | --- | --- |
| Cxcl14 | Cxcl14-F | CACTGCGAGGAGAAGATGG |
|  | Cxcl14-R | CACTTGATGAAGCGTTTGG |
| Pik3r5 | Pik3r5-F | TCTACTACTGCCGCTTTGC |
|  | Pik3r5-R | AGCCTTGATAGCCCGAGT |
| Timp1 | Timp1-F | TGCAACTCGGACCTGGAT |
|  | Timp1-R | TGGGACTTGTGGGCATATC |
| Lamc3 | Lamc3-F | CCGTCCCAGTGCCTACAA |
|  | Lamc3-R | TCAGTGCTGGTGACCCACT |
| PDGFα | Pdgfα-F | GATGAGGACCTGGGCTTG |
|  | Pdgfα-R | GATCAACTCCCGGGGTATCT |
| PDGFβ | Pdgfβ-F | CGAGGGAGGAGGAGCCTA |
|  | Pdgfβ-R | GTCTTGCACTCGGCGATTA |
| Ccl6 | Ccl6-F | TTATCCTTGTGGCTGTCCTTG |
|  | Ccl6-R | TGGAGGGTTATAGCGACGAT |
| Ccl9 | Ccl9-F | GATTGCTGCCTGTCCTAT |
|  | Ccl9-R | GTCCGTGGTTGTGAGTTT |
| Timp2 | Timp2-F | CGTTTTGCAATGCAGACGTA |
|  | Timp2-R | GGAATCCACCTCCTTCTCG |
| TGFβ2 | Tgfβ2-F | AGGAGGTTTATAAAATCGACATGC |
|  | Tgfβ2-R | TAGAAAGTGGGCGGGATG |
| Col3a1 | Col3a1-F | TCCCCTGGAATCTGTGAATC |
|  | Col3a1-R | TGAGTCGAATTGGGGAGAAT |
| Col1a2 | Col1a2-F | CAAGCATGTCTGGTTAGGAGAG |
|  | Col1a2-R | AGGACACCCCTTCTACGTTGT |
| Col1a1 | Col1a1-F | CATGTTCAGCTTTGTGGACCT |
|  | Col1a1-R | GCAGCTGACTTCAGGGATGT |
| Hapln4 | Hapln4-F | CAGCGTGGCAGGAAGAAAG |
|  | Hapln4-R | GGTAACGGCAGGGTAAGACAA |
| TGFβ1 | Tgfβ1-F | TGGAGCAACATGTGGAACTC |
|  | Tgfβ1-R | GTCAGCAGCCGGTTACCA |
| Timp3 | Timp3-F | CCTTTGGCACTCTGGTCT |
|  | Timp3-R | TCAGCAGGTACTGGTATTTGT |
| Pparα | Pparα-F | CTGAGACCCTCGGGGAAC |
|  | Pparα-R | AAACGTCAGTTCACAGGGAAG |
| Acox1 | Acox1-F | ATCAGGGCACCACTGCTC |
|  | Acox1-R | CCAAGCCTCGAAGATGAGTT |
| Cpt1b | Cpt1b-F | ATGTATCGCCGCAAACTG |
|  | Cpt1b-R | CCTGGGATGCGTGTAGTG |
| Fabp4 | Fabp4-F | GGATGGAAAGTCGACCACAA |
|  | Fabp4-R | TGGAAGTCACGCCTTTCATA |
| Ehhadh | Ehhadh-F | CCGGTCAATGCCATCAGT |
|  | Ehhadh-R | CTAACCGTATGGTCCAAACTAGC |
| Fabp3 | Fabp3-F | CTTTGTCGGTACCTGGAAGC |
|  | Fabp3-R | TGGTCATGCTAGCCACCTG |
| GAPDH | GAPDH-F | CAAGGTCATCCATGACAATTTG |
|  | GAPDH-R | GTCCACCACCCTGTTGCTGTAG |
| CD45 | CD45-F | ACATCATCGCCAGCATCTATC |
|  | CD45-R | CTTGCCTCCATCCACTTCATTA |
| CD163 | CD163-F | TGACGACAACTTCAGCAAAGA |
|  | CD163-R | CCAGAACCAGCTCCCAATTTA |
